# The predictive value of the neutrophil/eosinophil ratio in cancer patients undergoing immune checkpoint inhibition: a meta-analysis and a validation cohort in hepatocellular carcinoma

**DOI:** 10.3389/fimmu.2025.1633034

**Published:** 2025-07-21

**Authors:** Yang Xu, Yang Liu, Huimin Han, Zhen He, Wei Cao

**Affiliations:** Department of Oncology, Wuhan Third Hospital, Tongren Hospital of Wuhan University, Wuhan, Hubei, China

**Keywords:** immune checkpoint inhibitors, neutrophil-to-eosinophil ratio, prognosis, cancer, hepatocellular carcinoma

## Abstract

**Objective:**

This study was conducted to determine the prognostic relevance of neutrophil/eosinophil ratio (NER) in cancer patients receiving immune checkpoint inhibition therapy.

**Methods:**

A comprehensive search of the literature was carried out across PubMed, EMBASE, and the Cochrane Library to identify relevant studies published before May 2025. Key clinical endpoints included overall survival (OS), progression-free survival (PFS), objective response rate (ORR), and disease control rate (DCR). Additionally, a retrospective cohort analysis involving 67 hepatocellular carcinoma (HCC) patients who received ICIs at our center was undertaken to evaluate the prognostic significance of NER with respect to OS and PFS.

**Results:**

This meta-analysis incorporated 12 studies comprising a total of 1,716 patients. Higher baseline NER was consistently associated with poorer clinical outcomes, including shorter OS (HR = 1.82, 95% CI: 1.57–2.11, *p* < 0.001) and PFS (HR = 1.62, 95% CI: 1.34–2.97, *p* < 0.001), as well as lower ORR (HR = 0.50, 95% CI: 0.37–0.68, *p* < 0.001) and DCR (OR = 0.44, 95% CI: 0.31–0.61, *p* < 0.001). Complementing these findings, analysis of a retrospective cohort from our institution involving HCC patients revealed that individuals with higher NER experienced significantly worse OS (*p* = 0.006) and PFS (*p* = 0.033) when compared to those with lower NER levels.

**Conclusion:**

These findings underscore the prognostic significance of pretreatment NER in cancer patients receiving ICI therapy. Integrating NER into standard clinical evaluation may enhance risk stratification and contribute to the personalization of treatment strategies.

## 1 Introduction

Cancer remains the leading cause of death worldwide and continues to impose an increasingly severe threat to global health systems (1). The advent of monoclonal antibodies that inhibit immune checkpoints has ushered in a new era in oncology therapeutics (2, 3). Therapies based on immune checkpoint inhibitors (ICIs), particularly those targeting programmed cell death protein 1 (PD-1)/programmed death-ligand 1 (PD-L1) and cytotoxic T-lymphocyte–associated protein 4 (CTLA-4) pathways, have emerged as central pillars in modern immuno-oncology (4, 5). By reinvigorating immune responses or augmenting existing antitumor immunity, these approaches have shown substantial efficacy across a wide range of malignancies (6). Nonetheless, the clinical benefits are often limited to a subset of patients, and the absence of dependable predictive biomarkers remains a significant challenge (7). This highlights an urgent need to discover not only novel immunotherapeutic targets but also accessible, blood-derived biomarkers that can guide treatment selection. Such advances would expand the reach of ICI strategies and enhance their clinical impact across diverse cancer populations.

Neutrophils and eosinophils are both derived from myeloid progenitor cells but play distinct roles in the tumor microenvironment. Increasing evidence indicates a dynamic interplay between these two granulocyte populations (8, 9). Neutrophils often promote tumor progression through immunosuppressive mechanisms and facilitation of metastasis (8, 9), whereas eosinophils may exert anti-tumor effects by enhancing cytotoxic immune responses and secreting chemokines that recruit T cells. The NER, therefore, reflects a balance between pro-tumor and anti-tumor inflammatory forces. Given this biological rationale, NER has the potential to serve as an integrative prognostic biomarker, particularly in patients undergoing immune checkpoint inhibitor (ICI) therapy.

Emerging evidence suggests a potential link between a low baseline neutrophil/eosinophil ratio (NER) and favorable clinical outcomes in cancer patients receiving ICI therapy (10–12). In contrast, studies by Pozorski et al. and Zhuang et al. found no significant association between pretreatment NER and progression-free survival (PFS) in cancer patients (13, 14). To reconcile these contradictory results, the current study integrates both a meta-analytic framework and retrospective cohort analysis to comprehensively investigate the prognostic significance of NER in cancer patients treated with ICIs.

## 2 Methods

### 2.1 Literature search strategy, inclusion and exclusion criteria for the meta-analysis

Beginning in May 2025, a comprehensive electronic search was conducted across PubMed, EMBASE, and the Cochrane Library databases. The search utilized keywords such as “Neutrophil-to-Eosinophil Ratio” and “Neutrophil/Eosinophil Ratio.” The complete search syntax is available in the **Supplementary Material**. In addition to database retrieval, grey literature was reviewed via Google Scholar, and the reference lists of all eligible studies were manually screened for additional sources.

Studies were included if they met the following criteria: (1) enrolled patients diagnosed with cancer; (2) involved treatment with ICIs; (3) stratified patients into high and low NER groups; and (4) reported at least one relevant clinical endpoint—namely, overall survival (OS), PFS, objective response rate (ORR), or disease control rate (DCR). Studies were excluded if they were conference abstracts or commentary articles. When multiple publications reported on overlapping patient cohorts, only the version with the most complete dataset and rigorous methodology was included (15).

### 2.2 Data extraction and quality evaluation for the meta-analysis

Key information was systematically extracted from each eligible study, including the first author’s name, year of publication, study period, geographic location, tumor classification, treatment strategy, sample size, participant demographics (such as age and sex), and the cutoff value. When available, hazard ratios (HRs) from multivariate analyses were preferred over those from univariate models (16).

The methodological quality of the included observational studies was assessed using the Newcastle–Ottawa Scale (NOS) for cohort studies. This scale evaluates three broad domains: (1) Selection of study groups (up to 4 points), including representativeness of the exposed cohort, selection of the non-exposed cohort, ascertainment of exposure, and demonstration that outcome of interest was not present at the start of the study; (2) Comparability of cohorts based on design or analysis (up to 2 points); and (3) Outcome assessment (up to 3 points), including assessment of outcome, follow-up duration, and adequacy of follow-up. Studies scoring more than six points were considered high quality. All steps were performed independently by two reviewers. Any discrepancies between reviewers were resolved through consultation with the senior author.

### 2.3 Retrospective study cohort and data acquisition

This study received approval from the institutional ethics committee. Owing to its retrospective design, the requirement for informed consent was waived. A historical cohort analysis was conducted involving patients diagnosed with hepatocellular carcinoma (HCC) who underwent treatment with ICIs combined with anti-angiogenic agents at our center between Mar 2019 and May 2023. Inclusion criteria mandated at least one measurable tumor lesion as defined by RECIST version 1.1 (17).

Clinical and demographic information was extracted from electronic medical records and included patient age, gender, Eastern Cooperative Oncology Group performance status (ECOG PS), underlying hepatitis type, presence of cirrhosis, Barcelona Clinic Liver Cancer (BCLC) stage, Child–Pugh score, number of lesions, macrovascular invasion status, treatment line, modified albumin–bilirubin (mALBI) grade, and serum alpha-fetoprotein (AFP) levels. Tumor response and progression were evaluated according to RECIST version 1.1 criteria. Follow-up imaging via CT was routinely conducted at intervals of one to three months after treatment initiation. PFS was defined as the time from the first dose of immune checkpoint blockade to radiographic progression or death, while OS was measured from treatment initiation to death from any cause.

### 2.4 Statistical methods

Categorical data were expressed as absolute frequencies accompanied by their respective percentages. Survival outcomes across different subgroups were evaluated using the Kaplan–Meier estimator in conjunction with the Cox proportional hazards regression model. Meta-analytical computations were performed with Stata version 18.0, and the results were graphically summarized using forest plots. To quantify heterogeneity across included studies, both the I² statistic and Cochran’s Q test were applied. A heterogeneity level was considered significant if the I² exceeded 50% or the corresponding p-value was below 0.1 (18). When substantial variability was detected, the DerSimonian–Laird random-effects model was employed; otherwise, a fixed-effect model using the Inverse Variance method was applied.

Publication bias was investigated through Begg’s and Egger’s statistical tests (19). Sensitivity analyses were also conducted by sequentially omitting individual studies to assess the influence of each on the pooled HRs and overall effect estimates (20). Furthermore, subgroup analyses were conducted by stratifying data according to NER cutoff values and the type of Cox regression model used. A two-tailed p-value of less than 0.05 was considered to indicate statistical significance.

## 3 Results

### 3.1 Search results and study characteristics

**Figure 1:**
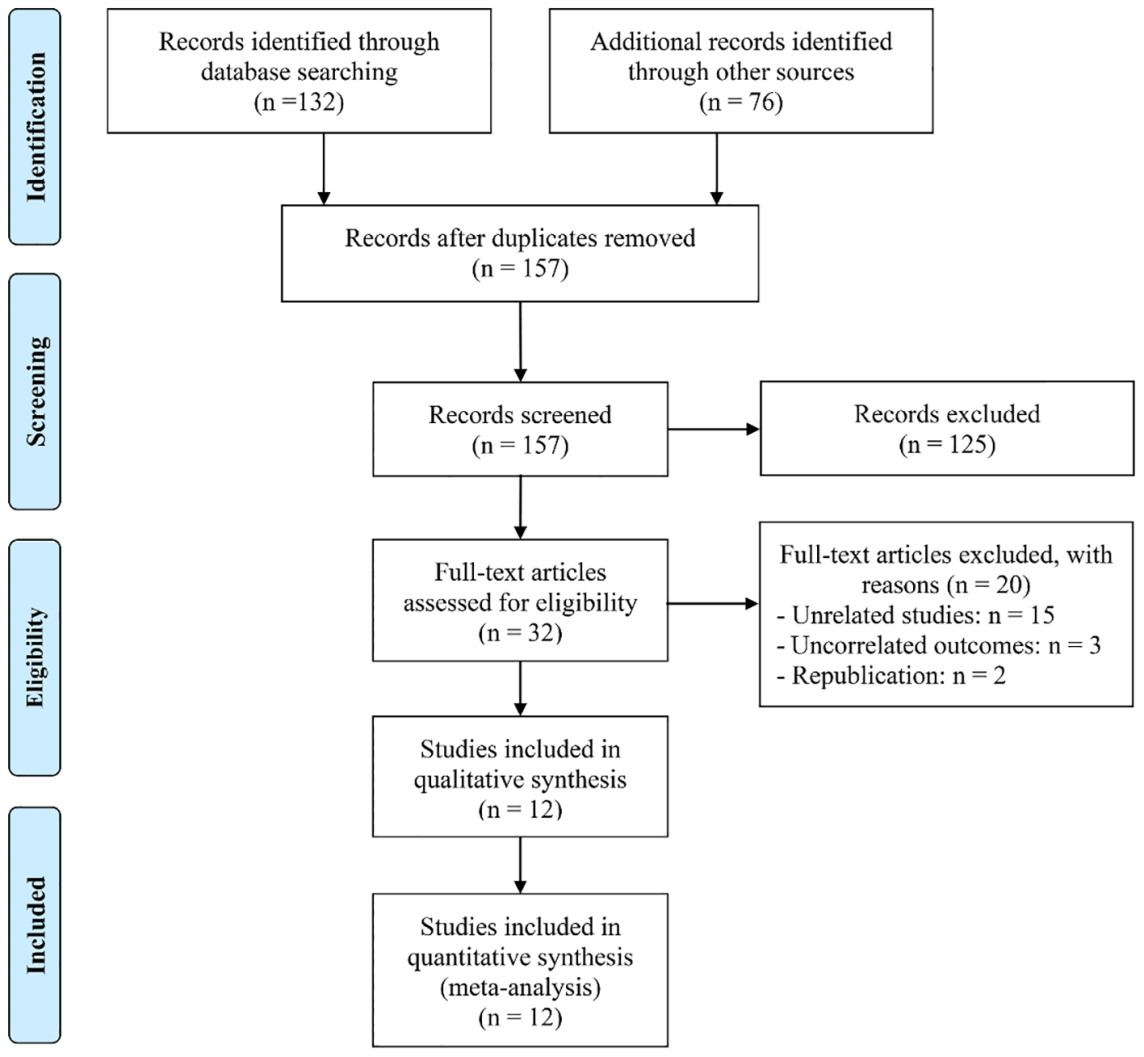
The flow diagram of identifying eligible studies.

An initial search across the databases, supplemented by manual screening of reference lists, yielded a total of 208 potentially relevant records. Following the removal of 54 duplicate entries, 125 studies were excluded after evaluation of titles and abstracts, as they did not meet the predefined inclusion criteria. A full-text assessment of the remaining 32 articles resulted in the exclusion of 20 papers that failed to satisfy the eligibility standards. Consequently, 12 studies were ultimately included in the meta-analysis (10–14, 21–27) (**Figure 1**).

**Table 1** provides an overview of the key characteristics of the included studies. In total, 1,716 individuals were enrolled, with sample sizes ranging from 21 to 401 per study. Of the 12 studies, five were conducted in the USA and two in Japan. Five studies involved patients with renal cell carcinoma, two with urothelial carcinoma, and two were pan-cancer studies. All studies employed a retrospective design. Based on the NOS, quality scores ranged from 6 to 8, indicating a low risk of bias (**Table 1**).

**Table 1:** Main characteristics of the studies included.

Study	Country	Cancer type	Treatment	Study period	Sample size	Age	Gender (male/female)	Cut‐point	NOS
Yildirim et al., 2025 (24)	USA	RCC	ICIs	01/2018-08/2023	401	66 (18–95)^b^	283/118	43.1	7
Pozorski et al., 2023 (14)	USA	Melanoma	Nivolumab or pembrolizumab	2011-2022	183	–	113/70	35.0	7
Tucker et al., 2024 (25)	USA	RCC	Avelumab plus axitinib or sunitinib	–	383	–	–	29.2	8
Zhuang et al., 2025 (13)	USA	pSCC	Nivolumab or pembrolizumab	2012-2023	21	56 (38–76)^b^	–	49.4	6
Gambale et al., 2024 (10)	Italian	UC	Avelumab	2021-2023	109	72 (54 -77)^a^	89/20	28.1	7
Suzuki et al., 2022 (22)	Japan	HNSCC	Nivolumab	10/2017-12/2021	47	67 (29–84)^b^	39/8	32	6
Beulque et al., 2024 (11)	UK, Belgium	RCC	Nivolumab with or without ipilimumab	2012-2022	201	67 (31-90)^b^	149/52	33.8	7
Liang et al., 2023 (21)	China	Pan-cancer	Anti‐PD‐(L)1	01/2019-12/2021	46	64 (57.5-67)^a^	41/6	18.43	7
Tucker et al., 2021 (26)	USA	RCC	Nivolumab plus ipilimumab	2016-2020	110	60.5 (54-69)^a^	84/26	26.4	7
Varayathu et al., 2021 (23)	India	Pan-cancer	Nivolumab or pembrolizumab	2017-2021	61	58^c^	42/19	24.3	7
Furubayashi et al., 2021 (27)	Japan	UC	Pembrolizumab	01/2018-06/2021	105	72 (67-77)^a^	75/30	13.7	7
Gil et al., 2022 (12)	Portugal	RCC	Nivolumab	06/2017-04/2021	49	61(28-85)^b^	42/7	48.0	6

^a^median (IQR), ^b^median (range), ^c^median. ICIs, immune checkpoint inhibitors; RCC, renal cell carcinoma; UC, uothelial carcinoma; HNSCC, head and neck squamous cell carcinoma; pSCC, penile squamous cell carcinoma.

### 3.2 Baseline neutrophil/eosinophil ratio and overall survival

**Figure 2:**
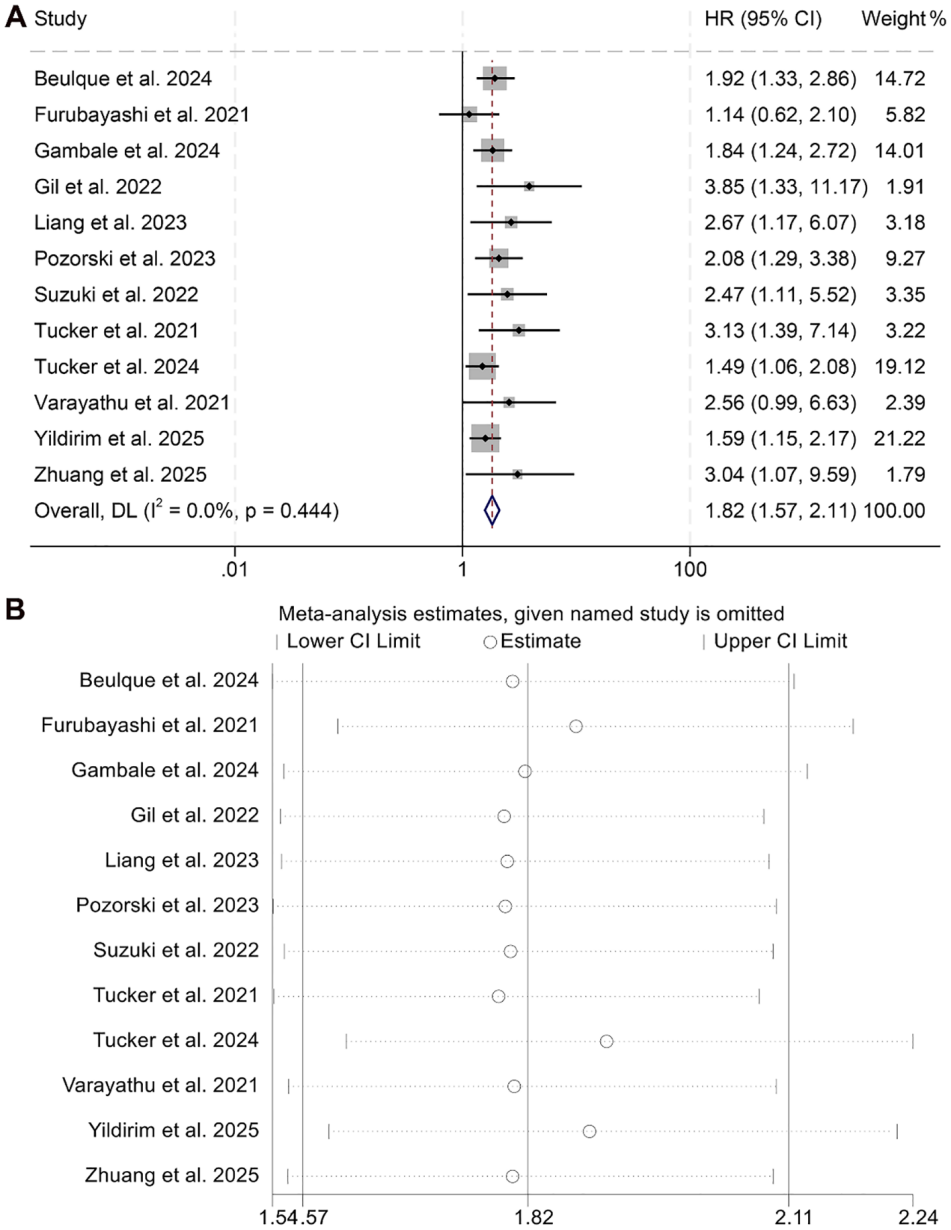
Forest plots depicting the association between the baseline neutrophil/eosinophil ratio and overall survival in cancer patients treated with ICIs **(A)**. Sensitivity analysis of the association between baseline neutrophil/eosinophil ratio and overall survival in cancer patients treated with ICIs **(B)**. HR, hazard ratio; CI, confidence interval.

This meta-analysis incorporated 12 qualified studies involving a total of 1,716 patients to assess the prognostic relevance of the NER on OS in individuals receiving ICI therapy. The aggregated HR indicated a significant association between higher NER levels and poorer OS outcomes (HR = 1.82, 95% CI: 1.57–2.11, *p* < 0.001; **Figure 2A**). Between-study variability was negligible, as reflected by Cochran’s Q test and an I² value (I² = 0, *p* = 0.444), supporting the use of a fixed-effects model.

Robustness of the pooled results was validated through sensitivity analysis, which involved the stepwise exclusion of each individual study. The overall estimates for OS remained consistent throughout this process (**Figure 2B**). Furthermore, assessments for publication bias using Begg’s and Egger’s tests revealed no statistically significant evidence of bias (Begg’s *p* = 0.118; Egger’s *p* = 0.108).

**Figure 3:**
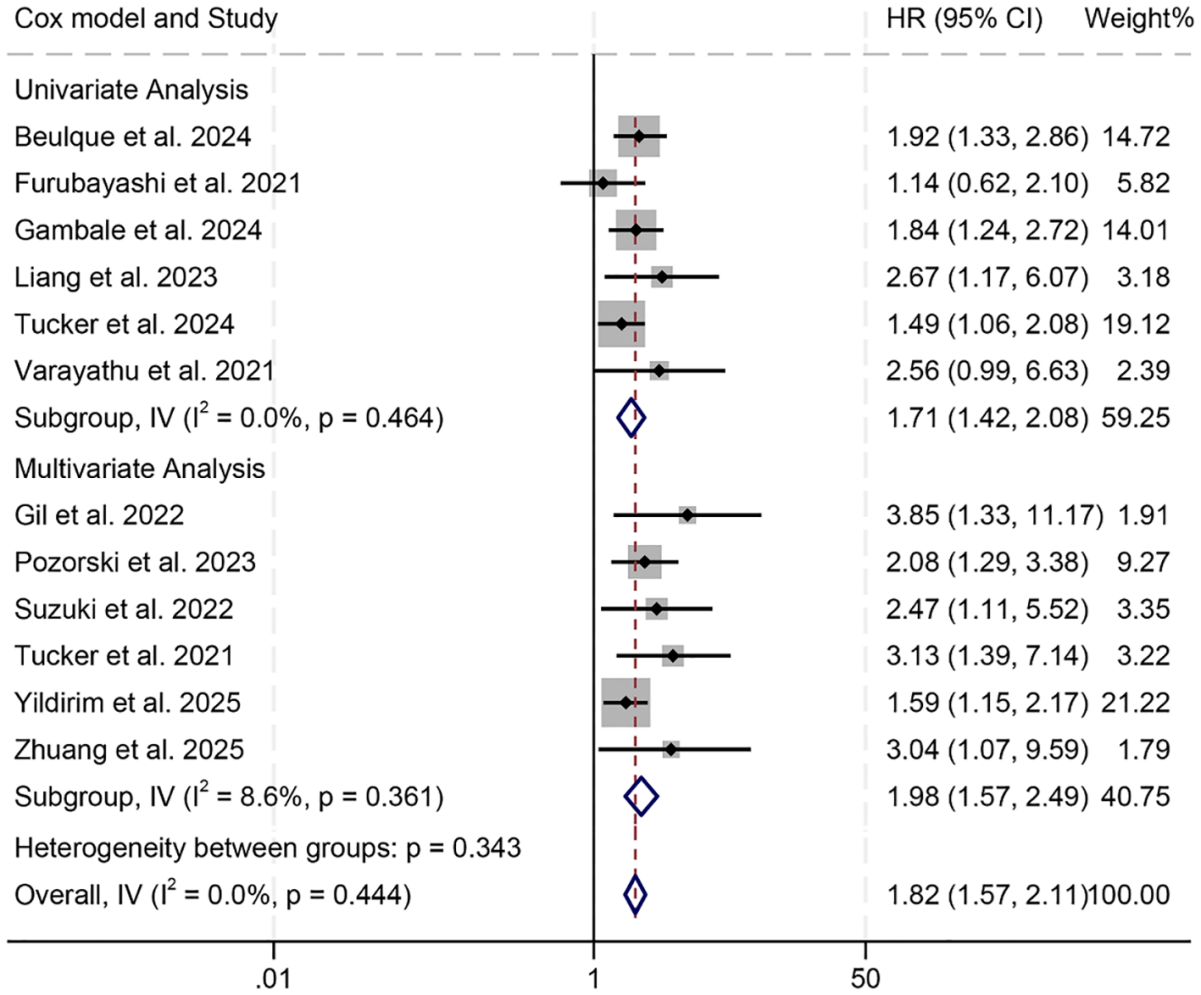
Subgroup analysis based on the Cox model revealed the relationship between the baseline neutrophil/eosinophil ratio and the overall survival of cancer patients treated with immune checkpoint inhibitors. HR, hazard ratio; CI, confidence interval.

Subgroup analyses demonstrated that both univariate (HR = 1.71, 95% CI: 1.42–2.08, *p* < 0.001) and multivariate regression approaches (HR = 1.98, 95% CI: 1.57–2.49, *p* < 0.001) consistently revealed a statistically significant link between elevated NER values and reduced OS (**Figure 3**). Moreover, this inverse association remained evident when the NER threshold exceeded 30 (HR = 1.91, 95% CI: 1.56–2.34, *p* < 0.001) or fell within the 20–30 range (HR = 1.81, 95% CI: 1.38–2.36, *p* < 0.001, **Supplementary Figure S1**). In contrast, when the cut-off point was below 20, NER failed to show prognostic value in predicting OS among cancer patients (HR = 1.67, 95% CI: 0.73–3.81, *p* = 0.227, **Supplementary Figure S1**).

### 3.3 Baseline neutrophil/eosinophil ratio and progression-free survival

**Figure 4:**
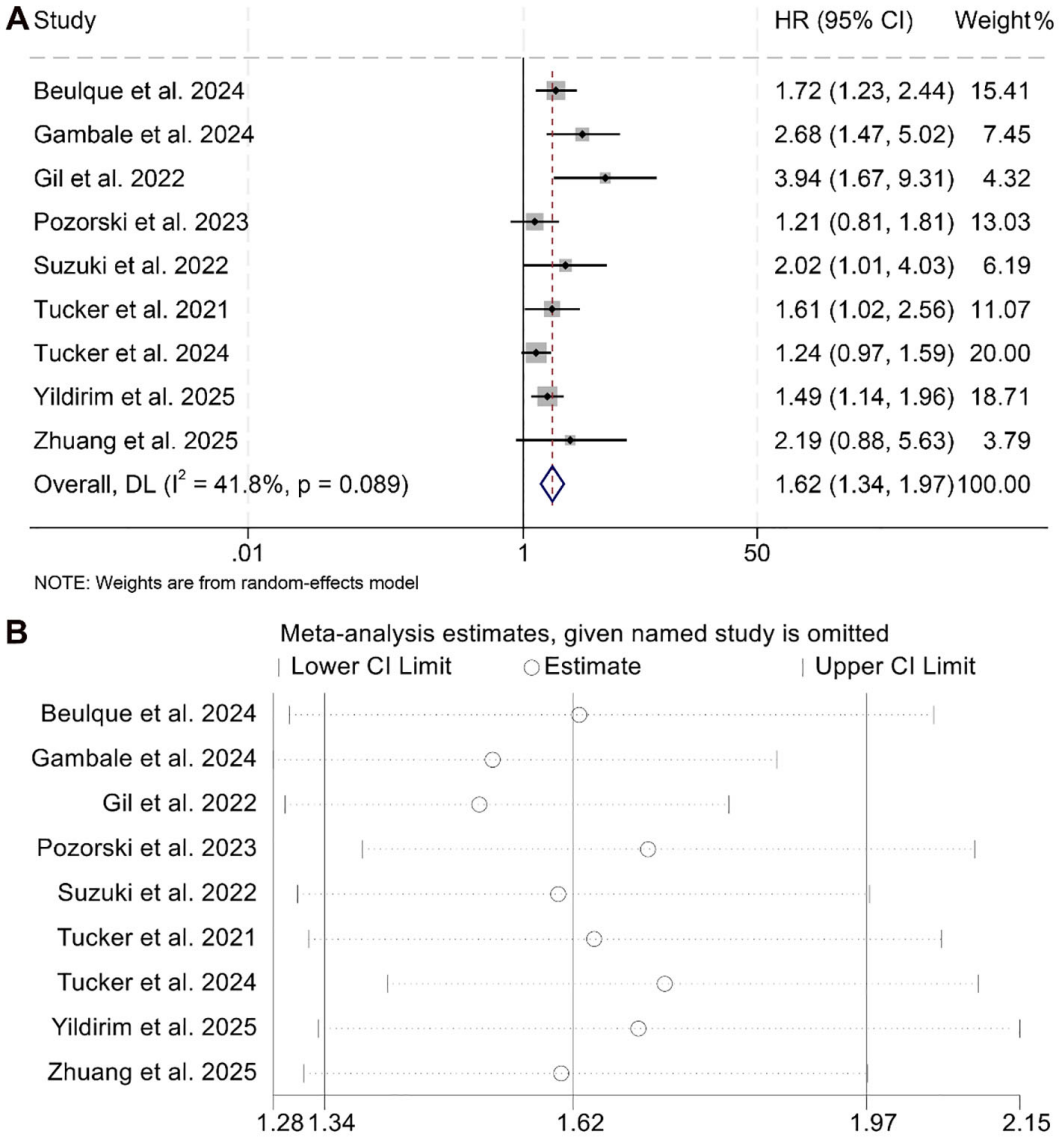
Forest plots depicting the association between the baseline neutrophil/eosinophil ratio and progression-free survival in cancer patients treated with ICIs **(A)**. Sensitivity analysis of the association between baseline neutrophil/eosinophil ratio and progression-free survival in cancer patients treated with ICIs **(B)**. HR, hazard ratio; CI, confidence interval. OR, odds ratio; CI, confidence interval.

This meta-analysis included nine eligible studies encompassing 1,504 patients to investigate the prognostic impact of the NER on PFS among individuals treated with ICIs. The pooled HR indicated a strong correlation between elevated NER and unfavorable PFS outcomes (HR = 1.62, 95% CI: 1.34–2.97, *p* < 0.001; **Figure 4A**). Substantial heterogeneity was observed among the studies (I² = 41.8%, *p* = 0.089), warranting the use of a random-effects model.

Sensitivity analysis, performed through sequential removal of each study, confirmed the stability of the combined PFS estimates (**Figure 4B**). In addition, assessments for potential publication bias using Begg’s and Egger’s tests did not indicate statistical evidence of asymmetry (Begg’s *p* = 0.129; Egger’s *p* = 0.145).

**Figure 5:**
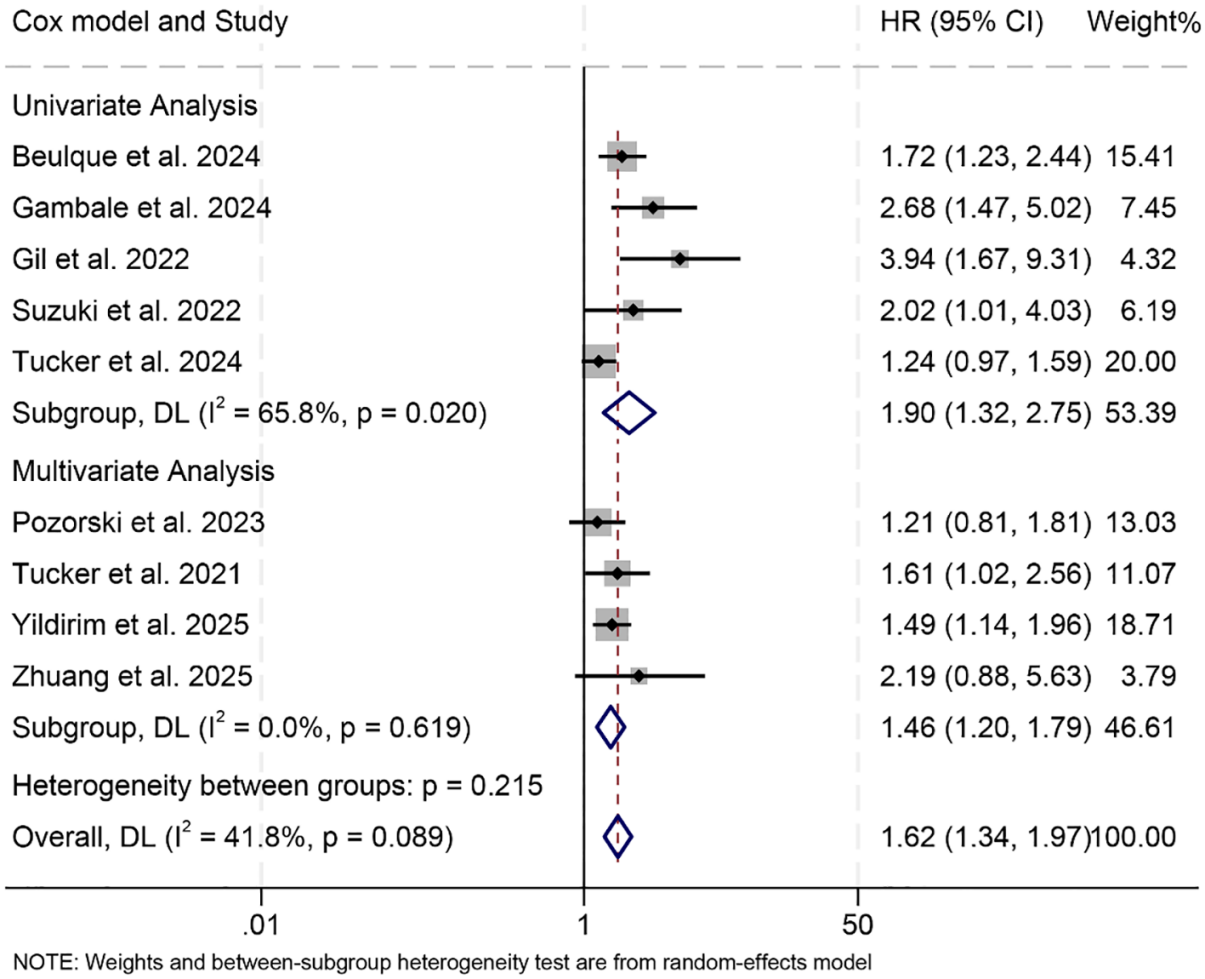
Subgroup analysis based on the Cox model revealed the relationship between the baseline neutrophil/eosinophil ratio and the progression-free survival of cancer patients treated with immune checkpoint inhibitors. HR, hazard ratio; CI, confidence interval. OR, odds ratio; CI, confidence interval.

Subgroup analyses further supported the association between high baseline NER and reduced PFS, with consistent results observed in both univariate (HR = 1.90, 95% CI: 1.32–2.75, *p* < 0.001) and multivariate models (HR = 1.46, 95% CI: 1.20–1.79, *p* < 0.001, **Figure 5**). Notably, this negative prognostic relationship was maintained when the NER cut-off was greater than 30 (HR = 1.66, 95% CI: 1.31–2.10, *p* < 0.001) or within the 20–30 range (HR = 1.62, 95% CI: 1.07–2.45, *p* < 0.001, **Supplementary Figure S2**).

### 3.4 Baseline neutrophil/eosinophil ratio and objective response rate

**Figure 6:**
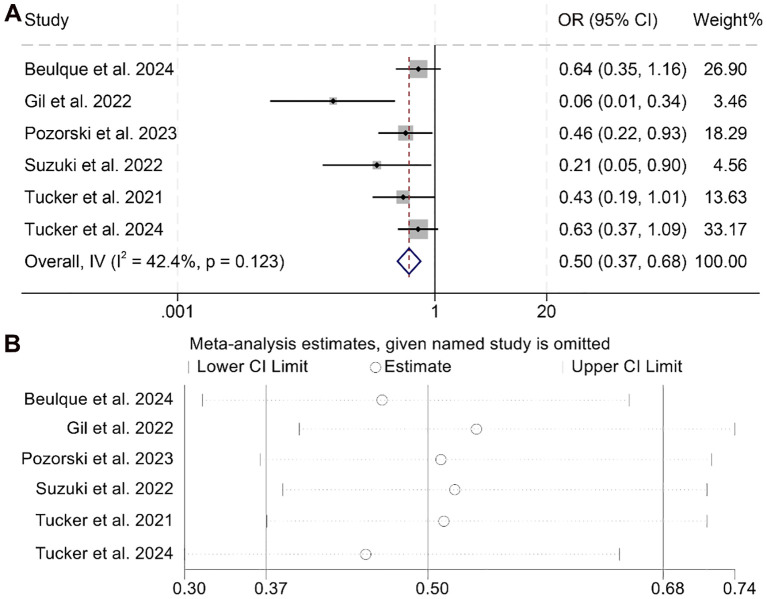
Forest plots depicting the association between the baseline neutrophil/eosinophil ratio and objective response rate in cancer patients treated with ICIs **(A)**. Sensitivity analysis of the association between baseline neutrophil/eosinophil ratio and objective response rate in cancer patients treated with ICIs **(B)**. OR, odds ratio; CI, confidence interval.

Our study further investigated the association between NER and ORR, incorporating data from six studies involving a total of 973 cancer patients. As no significant heterogeneity was observed across these studies, a fixed-effect model was applied (I² = 42.4%, *p* = 0.123). The meta-analysis demonstrated that patients with elevated NER had a significantly lower ORR compared to those in the low-NER group (OR = 0.50, 95% CI: 0.37–0.68, *p* < 0.001; **Figure 6A**).

Sensitivity analysis confirmed the robustness of this finding, as sequential exclusion of individual studies did not materially alter the overall effect estimate (**Figure 6B**). Assessments for publication bias using Begg’s and Egger’s tests revealed no statistically significant evidence of bias (Begg’s *p* = 0.328; Egger’s *p* = 0.428). Moreover, subgroup analysis based on different NER cut-off values consistently supported the observed association, indicating that the inverse relationship between NER and ORR remained stable across various threshold definitions (**Supplementary Figure S3**).

### 3.5 Baseline neutrophil/eosinophil ratio and disease control rate

**Figure 7:**
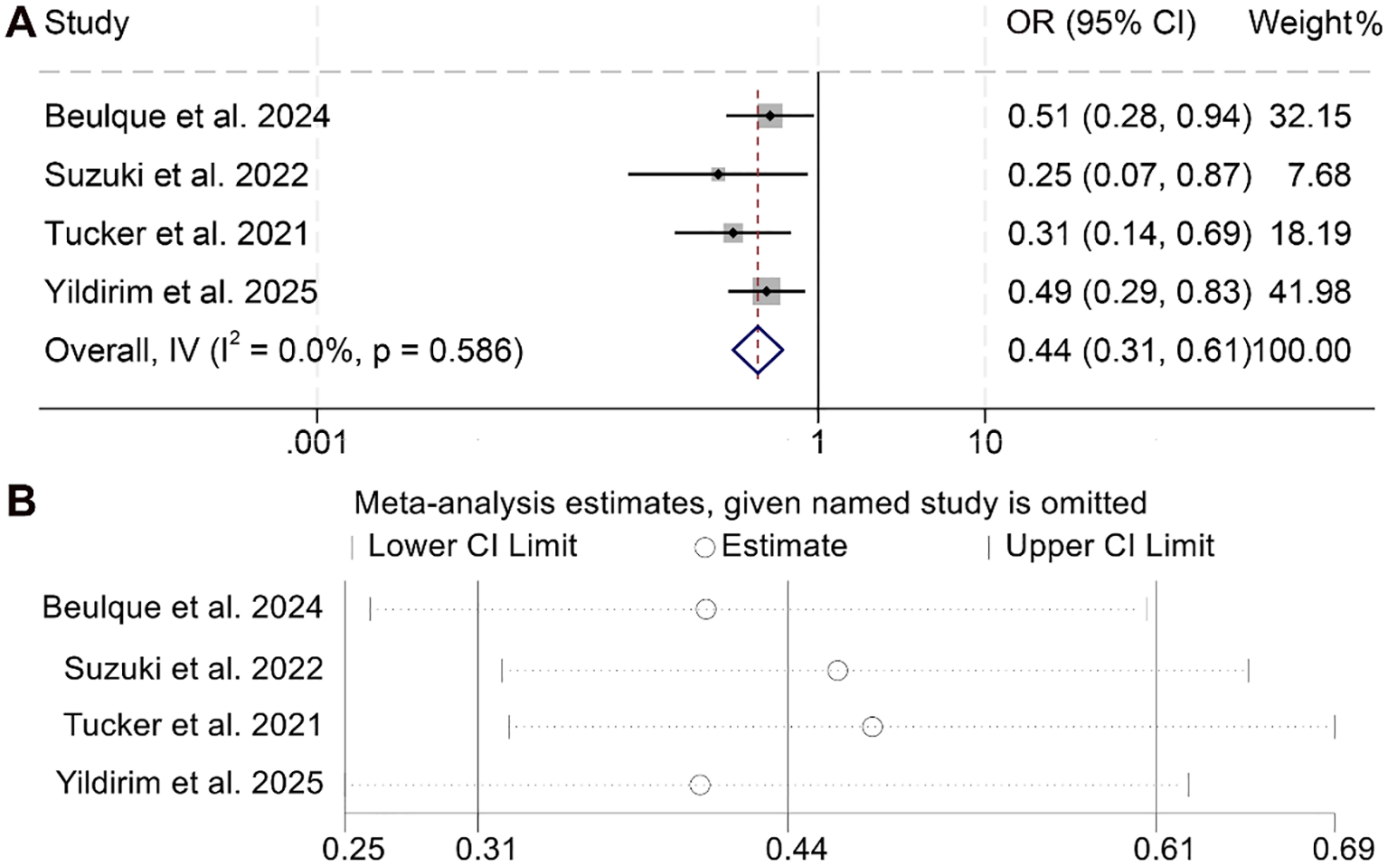
Forest plots depicting the association between the baseline neutrophil/eosinophil ratio and disease control rate in cancer patients treated with ICIs **(A)**. Sensitivity analysis of the association between baseline neutrophil/eosinophil ratio and disease control rate in cancer patients treated with ICIs **(B)**. OR, odds ratio; CI, confidence interval.

The relationship between NER and DCR among cancer patients was analyzed based on four studies comprising 759 individuals. As the analysis revealed no significant heterogeneity (I² = 0, *p* = 0.586), a fixed-effects model was deemed appropriate. The aggregated findings demonstrated that higher NER levels were significantly correlated with a lower DCR compared to patients with lower NER values (OR = 0.44, 95% CI: 0.31–0.61, *p* < 0.001; **Figure 7A**).

To assess the reliability of these results, sensitivity analyses were performed by systematically excluding each study. The consistency of the effect estimates across iterations confirmed the robustness and stability of the pooled outcome for DCR (**Figure 7B**).

### 3.6 Prognostic role of neutrophil/eosinophil ratio in our HCC cohort

In view of the limited literature addressing the prognostic relevance of the NER in hepatocellular carcinoma (HCC), we conducted an analysis using patient data from our institution to enhance current insights into NER as a prognostic biomarker in oncology.

**Supplementary Table 1** presents the baseline demographic and clinical profiles of the 67 patients with HCC included in our cohort. The median age was 58.2 years, with an age range spanning from 40.2 to 81.23 years. A predominance of male participants was observed, accounting for 67.16% (n = 45) of the total. Regarding performance status, 62.69% (n = 42) had an ECOG PS score of 0, while the remaining 37.31% (n = 25) had a score of 1. Chronic viral hepatitis was documented in 77.61% (n = 52) of the cohort, and cirrhosis of the liver was present in 67.16% (n = 45). According to the BCLC staging system, 5.97% (n = 4) were categorized as early stage, 41.79% (n = 28) as intermediate stage, and 52.24% (n = 35) as advanced stage. Microvascular invasion was identified in 35.82% (n = 24) of patients, and AFP levels exceeded 400 ng/mL in 58.21% (n = 39) of cases.

**Figure 8:**
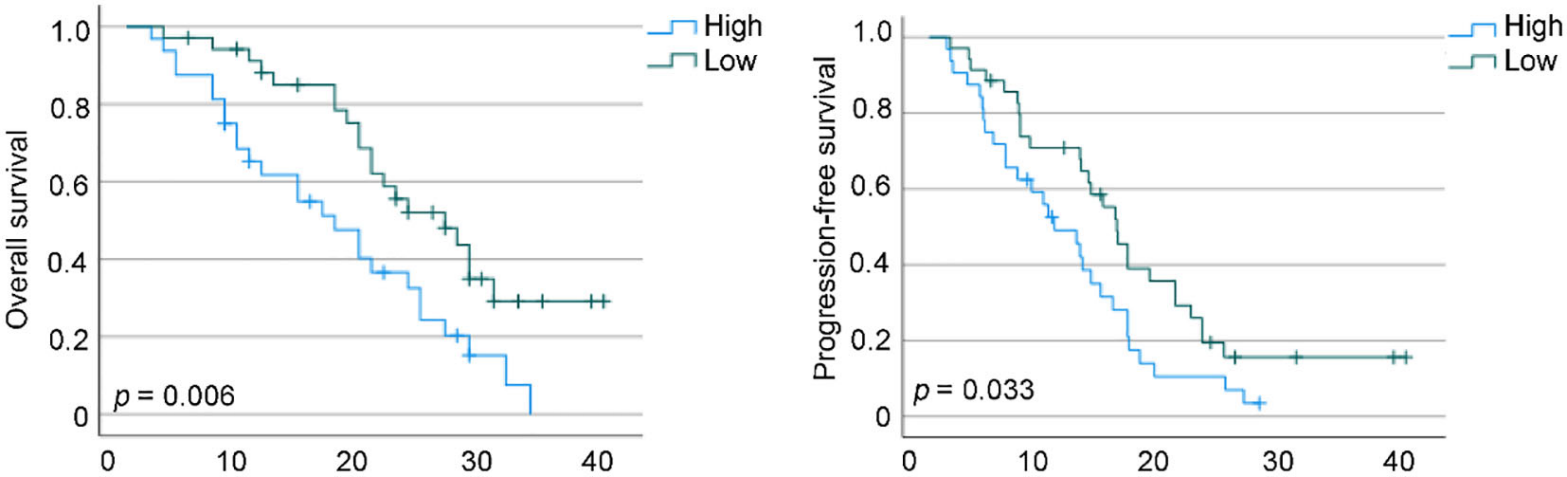
Kaplan–Meier survival estimates for overall survival and progression-free survival are presented, stratified by baseline neutrophil-to-eosinophil ratio levels in our cohorts. HR, hazard ratio; CI, confidence interval.

Patients were stratified into two subgroups according to the median baseline NER threshold. Kaplan–Meier analysis demonstrated that individuals with higher NER exhibited markedly reduced OS (*p* = 0.006) as well as PFS (*p* = 0.033) when compared to those with lower NER values (**Figure 8**).

## 4 Discussion

As a biomarker easily obtainable through routine hematological testing, the NER offers a cost-efficient and widely accessible measure. In this study, elevated baseline NER was significantly associated with worse survival outcomes among cancer patients. Furthermore, its prognostic relevance remained robust across varying regression models and stratifications based on different threshold definitions.

Earlier investigations have demonstrated that increased circulating neutrophil counts, along with their accumulation within the tumor microenvironment, are linked to unfavorable responses to immune checkpoint blockade therapies (8, 9). Tumor-associated neutrophils (TANs) contribute to poor clinical outcomes by promoting processes such as aerobic glycolysis, angiogenic signaling, formation of neutrophil extracellular traps (NETs), and activation of immunosuppressive mechanisms (28–31).

While eosinophils are well-recognized for their involvement in allergic conditions, parasitic infections, and certain viral responses, their functions within the tumor microenvironment (TME) remain comparatively underexplored relative to other immune cell subsets (32). A growing body of literature has underscored their role in tumor progression and metastasis. *In vitro* experiments suggest that eosinophils contribute to tumor cell eradication by engaging in complex cellular interactions with B lymphocytes, Th1/Th2 CD4^+^ T cells, and other granulocytes (33). Upon encountering tumor-associated molecular signatures and receiving cues from the immune milieu, eosinophils undergo degranulation, releasing a spectrum of effector molecules—such as TNF-α, granzymes, major basic protein (MBP), and metalloproteinases—that facilitate immune cell recruitment, enhance antigen presentation, and directly induce tumor cytotoxicity (34, 35).

Moreover, eosinophils secrete ribonucleases and cationic proteins capable of forming extracellular traps that promote tumor cell lysis. *In vivo* models have demonstrated that CC-chemokines play a critical role in guiding eosinophils into tumors and enhancing their cytotoxic activity. Chemokines such as C-C motif chemokine ligand 5 (CCL5), C-C motif chemokine ligand 11 (CCL11), C-X-C motif chemokine ligand 9 (CXCL9), and C-X-C motif chemokine ligand 10 (CXCL10) are believed to be primary mediators of eosinophil-driven tumor necrosis. Notably, diminished expression of CCL11 has been linked to increased tumor load and reduced eosinophil infiltration in preclinical mouse models (36, 37).

Previous studies using melanoma models indicate that tumor cell apoptosis itself may serve as a recruitment signal for eosinophils. Although the precise mechanisms remain to be elucidated, retrospective clinical evidence in melanoma has hinted at a favorable association between lower baseline NER or elevated eosinophil counts and enhanced response to first-line immunotherapy (38, 39). These mechanistic insights offer strong biological support for the findings observed in our present study.

In addition to NER, several other peripheral blood-based biomarkers have shown potential in predicting outcomes in cancer patients undergoing immune checkpoint inhibitor therapy (15, 28, 35, 37, 40). These include the neutrophil-to-lymphocyte ratio (NLR), platelet-to-lymphocyte ratio (PLR), and systemic immune-inflammation index (SII), all of which reflect systemic inflammation and immune status (28). For instance, elevated NLR has been associated with worse prognosis in various malignancies treated with ICIs (28, 35). While these markers may offer complementary insights, there is currently no consensus on which parameter provides the most reliable predictive value. Future prospective studies are needed to directly compare the prognostic performance of NER with these alternative indices and to determine their utility in composite prognostic models.

Although this meta-analysis provides valuable insights, several inherent limitations must be acknowledged. Most notably, the analysis is based solely on retrospective cohort studies, which may compromise the robustness and accuracy of the pooled estimates. Additionally, variation in the definition of NER cut-off values across the included studies introduces methodological inconsistencies. To address these concerns, future investigations should focus on prospective, multicenter trials employing harmonized protocols, thereby improving the generalizability and clinical applicability of NER as a prognostic indicator in oncology.

## 5 Conclusion

These findings underscore the prognostic utility of the pretreatment NER in cancer patients receiving immune checkpoint inhibitors. As a readily accessible and cost-effective biomarker, NER could be integrated into standard oncology workflows to support pre-treatment risk stratification. Future prospective studies are warranted to validate standardized NER thresholds and to assess its integration into clinical decision-making algorithms for optimizing immunotherapy outcomes.

## Data Availability

The original contributions presented in the study are included in the article/**Supplementary Material**. Further inquiries can be directed to the corresponding authors.
