# Supplementary material for: The predictive value of the neutrophil/eosinophil ratio in cancer patients undergoing immune checkpoint inhibition: a meta-analysis and a validation cohort in hepatocellular carcinoma
Source: Front Immunol. 2025 Jul 21;16:1633034. doi: 10.3389/fimmu.2025.1633034 (PMC12318750; doi:10.3389/fimmu.2025.1633034)

**Supplementary material 1. Literature retrieval strategy**

(((((Neutrophil-to-Eosinophil Ratio) or (Neutrophil to Eosinophil Ratio)) OR (Neutrophil-Eosinophil Ratio)) OR (Neutrophil/Eosinophil Ratio)) OR (NER)) AND ((Camrelizumab) OR (Sintilimab) OR (Tislelizumab) OR (Toripalimab) OR (Envafolimab) OR (Immune Checkpoint Inhibitors) OR (Checkpoint Inhibitors, Immune) OR (Immune Checkpoint Inhibitor) OR (Checkpoint Inhibitor, Immune) OR (Immune Checkpoint Blockers) OR (Checkpoint Blockers, Immune) OR (Immune Checkpoint Blockade) OR (Checkpoint Blockade, Immune) OR (Immune Checkpoint Inhibition) OR (Checkpoint Inhibition, Immune) OR (PD-L1 Inhibitors) OR (PD L1 Inhibitors) OR (PD-L1 Inhibitor) OR (PD L1 Inhibitor) OR (Programmed Death-Ligand 1 Inhibitors) OR (Programmed Death Ligand 1 Inhibitors) OR (PD-1-PD-L1 Blockade) OR (Blockade, PD-1-PD-L1) OR (PD 1 PD L1 Blockade) OR (CTLA-4 Inhibitors) OR (CTLA 4 Inhibitors) OR (CTLA-4 Inhibitor) OR (CTLA 4 Inhibitor) OR (Cytotoxic T-Lymphocyte-Associated Protein 4 Inhibitors) OR (Cytotoxic T Lymphocyte Associated Protein 4 Inhibitors) OR (Cytotoxic T-Lymphocyte-Associated Protein 4 Inhibitor) OR (Cytotoxic T Lymphocyte Associated Protein 4 Inhibitor) OR (PD-1 Inhibitors) OR (PD-1 Inhibitor) OR (PD 1 Inhibitors) OR (Inhibitor, PD-1) OR (PD 1 Inhibitor) OR (Programmed Cell Death Protein 1 Inhibitor) OR (Programmed Cell Death Protein 1 Inhibitors) OR (Pembrolizumab) OR (Nivolumab) OR (Atezolizumab) OR (Ipilimumab) OR (Avelumab) OR (Tremelimumab) OR (Durvalumab) OR (Cemiplimab) OR (Immune Checkpoint Inhibitors[MeSH Terms]))

| Supplementary Table 1. Patient characteristics | |
| --- | --- |
|  | Overall (n=67) |
| Age | 58.2 (40.2-81.23) |
| Males | 45 (67.16%) |
| ECOG PS |  |
| 0 | 42 (62.69%) |
| 1 | 25 (37.31%) |
| Etiology |  |
| Viral | 52 (77.61%) |
| Other | 15 (22.39%) |
| Liver cirrhosis |  |
| Yes | 45 (67.16%) |
| No | 22 (32.84%) |
| BCLC stage |  |
| Early | 4 (5.97%) |
| Intermediate | 28 (41.79%) |
| Advanced | 35 (52.24%) |
| Child-Pugh class |  |
| A | 52 (77.61%) |
| B | 15 (22.39%) |
| Tumor number |  |
| < 3 | 51 (76.12%) |
| ≥ 3 | 16 (23.88%) |
| Macrovascular invasion |  |
| Yes | 24 (35.82%) |
| No | 43 (64.18%) |
| Treatment line |  |
| First-line | 40 (59.70%) |
| Later-line | 27 (40.30%) |
| mALBI grade |  |
| 1 | 30 (44.78%) |
| 2 | 37 (55.22%) |
| AFP (ng/mL) |  |
| ≥ 400 | 39 (58.21%) |
| < 400 | 28 (41.79%) |
| Data shown are means with range or numbers with percentage.  ECOG PS, Eastern Cooperative Oncology Group performance status; BCLC, Barcelona Clinic Liver Cancer; AFP, α-fetoprotein; mALBI grade, modified albumin-bilirubin grade. | |

Figure S1. Subgroup analysis based on the cut-off revealed the relationship between the baseline neutrophil/eosinophil ratio and the overall survival of cancer patients treated with immune checkpoint inhibitors. HR, hazard ratio; CI, confidence interval.


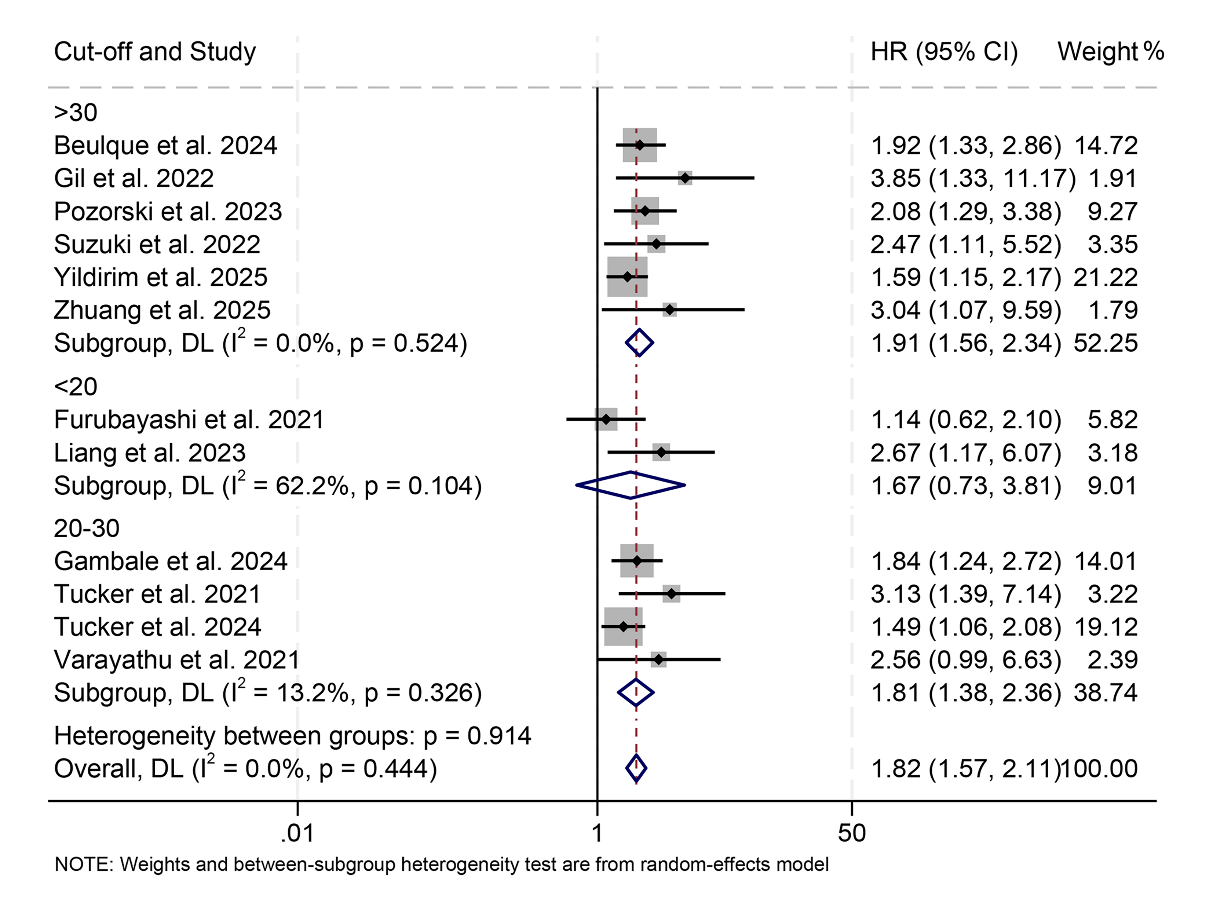


Figure S2. Subgroup analysis based on the cut-off revealed the relationship between the baseline neutrophil/eosinophil ratio and the progression-free survival of cancer patients treated with immune checkpoint inhibitors. HR, hazard ratio; CI, confidence interval.


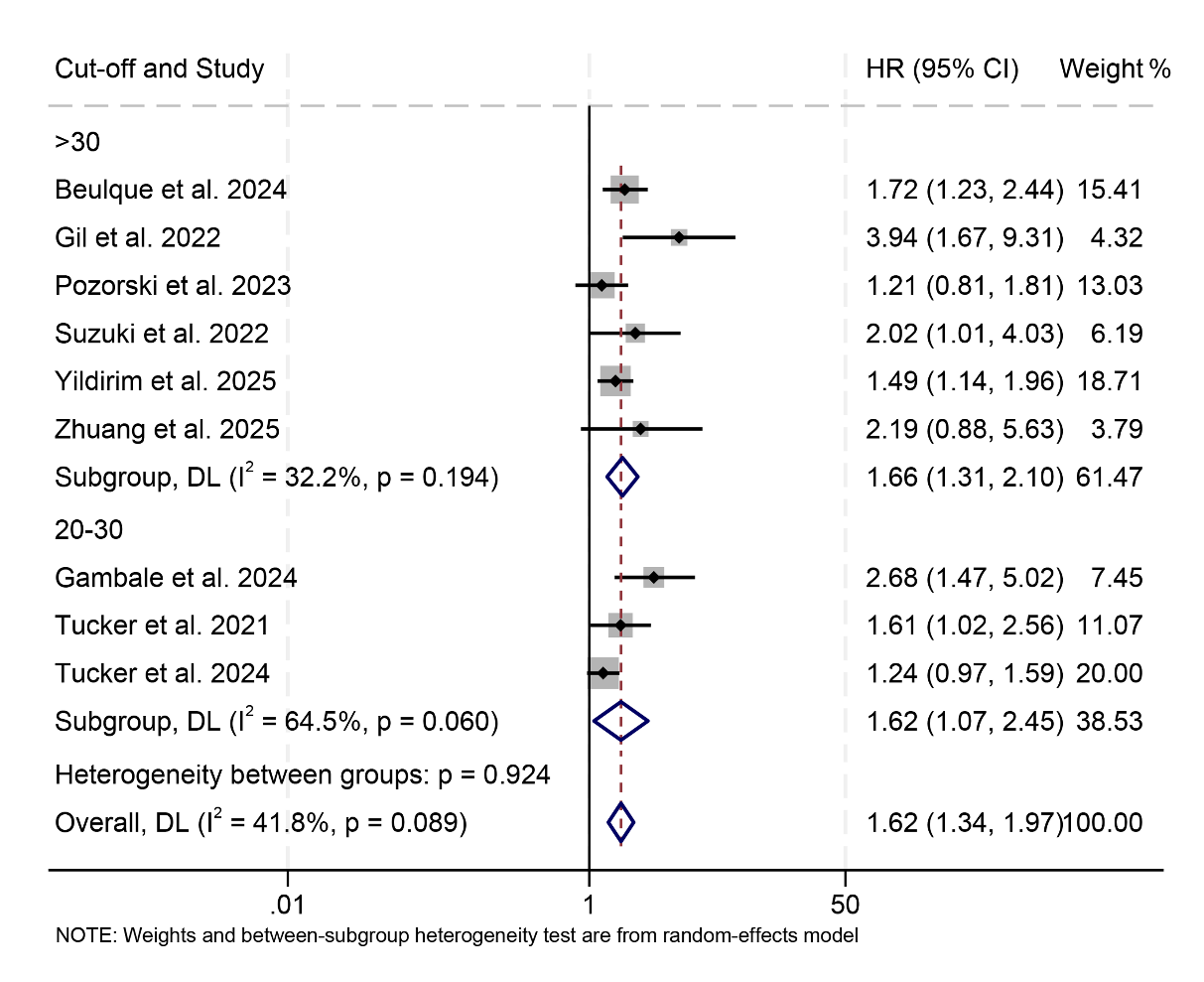


Figure S3. Subgroup analysis based on the cut-off revealed the relationship between the baseline neutrophil/eosinophil ratio and thebjective response rate of cancer patients treated with immune checkpoint inhibitors. OR, odds ratio; CI, confidence interval.


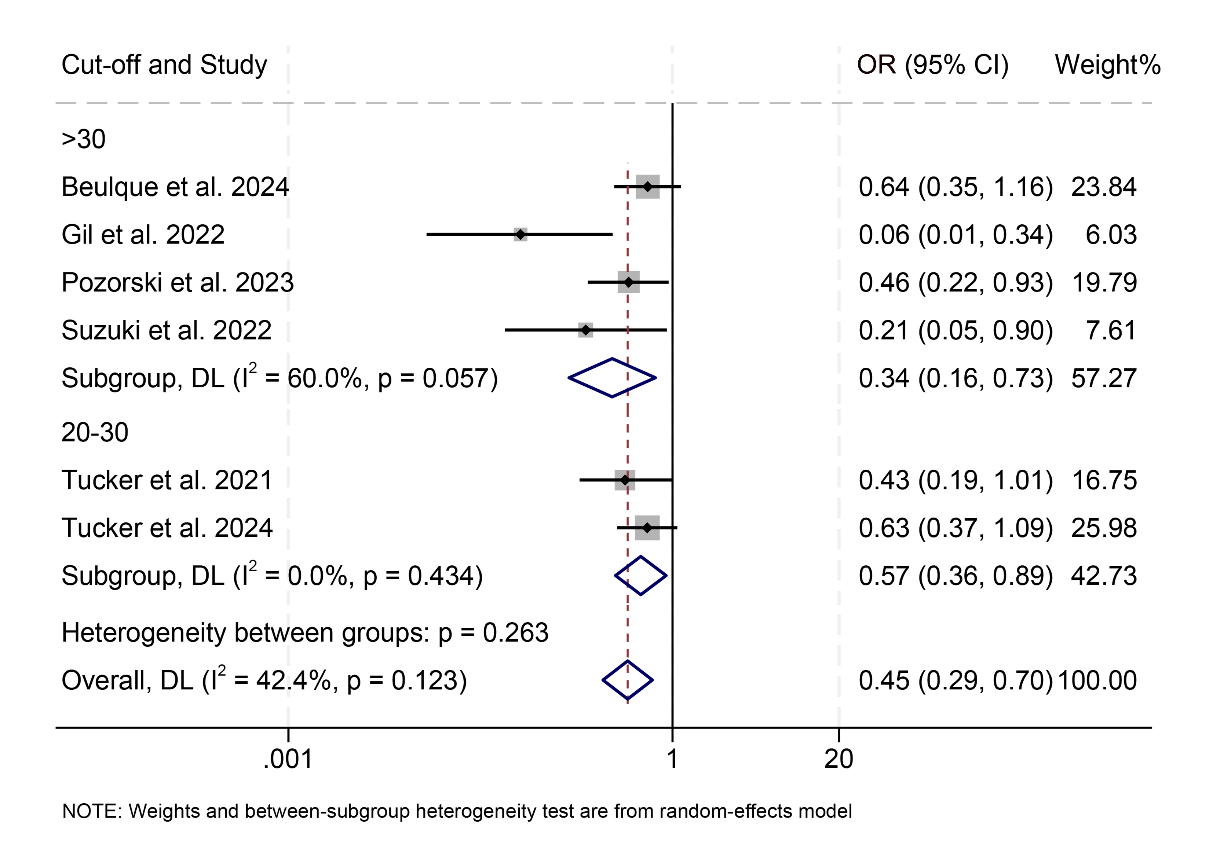

Supplement: Supplementary file 1 [file DataSheet1.docx]
